# The psychological impact of COVID-19 on university students in China and Africa

**DOI:** 10.1371/journal.pone.0270824

**Published:** 2022-08-04

**Authors:** Pamela Marahwa, Panashe Makota, Donald Tafadzwa Chikomo, Tawanda Chakanyuka, Tsitsi Ruvai, Kelvin Stefan Osafo, Tianwen Huang, Limin Chen

**Affiliations:** 1 Department of Respiratory Medicine, Fujian Medical University Union Hospital, Fuzhou, Fujian, People’s Republic of China; 2 Fujian Provincial Hospital, Fujian Medical University, Fuzhou, Fujian, People’s Republic of China; 3 Fujian Medical University Union Hospital, Fuzhou, Fujian, People’s Republic of China; 4 The First Affiliated Hospital of Fujian Medical University, Fuzhou, Fujian, People’s Republic of China; 5 Department of Neurology, Fujian Medical University Union Hospital, Fuzhou, Fujian, People’s Republic of China; 6 Fujian Key Laboratory of Vascular Aging (Fujian Medical University), Fuzhou, Fujian, People’s Republic of China; Flinders University, AUSTRALIA

## Abstract

The COVID-19 pandemic is alarmingly a global health catastrophe that has created an unprecedented mental health decline especially in young adults, who have been noted to be a vulnerable population. In this study, we investigated the prevalence of depression and anxiety in university students in China and Africa during the COVID-19 pandemic, the significant factors contributing to the prevalence of anxiety and depression, the differences in factors affecting the different groups being investigated and to emphasize that psychological intervention are as important as the physical interventions during and after the pandemic. The study was conducted through online surveys, with 684 participants using Patient Health Questionnaire-9 and Generalized Anxiety Disorder-7 standardized scales. Comparing all groups combined, of the 636 participants, 361 (56.8%) had depression and 227 (35.7%) had anxiety. Chi squared tests at significance level (P<0.05) showed that country of citizenship, religion, parents’ educational background, household monthly income and, having family members with COVID-19 variables were strongly associated with depression and anxiety. In contrast, age, gender, educational background, and major showed no significant association. Comparing the individual groups separately using chi square (P<0.05), the Chinese students in China group had 35.6% with depression and 13.1% with anxiety. The variable associated with both depression and anxiety was education major, with depression only was parent’s educational background and with anxiety only was gender. The African students in China group had 70.3% with depression and 45.0% with anxiety. Gender was strongly associated with both depression and anxiety, and religion and having family members with COVID-19 with anxiety only. Africans in Africa had 66.0% with depression and 50.5% with anxiety. Educational background was strongly associated with depression. There was no statistically significant variable for anxiety. Chi square test showed a statistically significant difference in depression and anxiety levels with the Chinese group compared to both African groups, and no significant difference between both African groups. Our findings demonstrated that COVID-19 had a negative psychological impact on university students. Therefore, more attention should be put on youth’s mental health during this pandemic.

## Introduction

COVID-19 is a highly infectious respiratory disease caused by severe acute respiratory syndrome coronavirus 2 (SARS-COV-2) [1]. It was first identified in Wuhan City, Hubei Province, China in December 2019 and declared by WHO a global pandemic in March 2020 [2]. SARS-COV-2 is transmitted through respiratory droplets, close contact, and asymptomatic carriers. Symptoms appear after an incubation period between 2–14 days post-exposure and range from asymptomatic to severe pneumonia. Some of the clinical manifestations include dry cough, fever, dyspnea, muscle aches, etc. Other studies portray that patients with underlying medical conditions such as cardiovascular diseases, diabetes, chronic respiratory diseases, cancer, and old-age people are more likely to experience severe morbidity [3–6]. As of 25th July 2021, there were over 194 million confirmed cases and 4.15 million confirmed deaths globally [7]. In efforts to curb the spread of the virus, the government and national health commissions have implemented infection control measures which included movement restrictions, confinement to homes, closure of schools, isolation, quarantine, wearing of masks, social distancing, and personal hygiene which includes washing of hands and the use of sanitizers [8]. Although these measures have been effective in preventing the transmission of COVID-19, concerns have arisen about the psychological impact isolation and quarantine have on individuals [9].

In reference to previous pandemics, studies have highlighted that there is a surge in mental catastrophes during and post-pandemics. In 2003, a SARS outbreak similar to the ongoing crisis, emerged affecting countries such as China, Singapore, Hong Kong & Taiwan. A study conducted in Hong Kong 30 months post-SARS crisis reflected that the cumulative incidence of psychiatric disorders was at 33.3%, of which 25% of the patients had PTSD and 15.6% a depressive disorder. SARS, being limited to a small area, yielded the above statistics, it can only be inferred that COVID-19, being a global crisis, affecting millions of lives and causing more deaths, will present with more psychological complications. Therefore, a corresponding global mental crisis is on the rise, hence a need to consider more effort to address a fast-approaching psychological tragedy [9, 10].

During this pandemic, some studies have shown its psychological effects varying from panic to pervasive feelings of hopelessness and desperation which may lead to suicide [11]. A survey of 130 countries conducted by WHO showed that the pandemic has caused a devastating mental health crisis on the general population and urgent need for critical mental health services [12], with younger people presenting with higher levels. This came as a result of quarantine and isolation, cessation of travel, the uncertainty of the future, economic crisis, fear of contracting the virus, insufficient information and supplies, boredom, loneliness, stigma, etc [13]. Multiple studies have also highlighted the association between COVID-19, mental health and suicidal behavior, showing an increase in suicide rates with increase in mental health cases during the pandemic [14] Moreover, internet suicide-related search volumes demonstrated that some individuals that commit suicide sought information online, during the pandemic [15]. Depression was cited as one of the major factors leading to recorded suicidal attempts with anxiety mentioned alongside [16]. Despite this evidence, research studies have focused more on the COVID-19 disease and its patients, and less on its psychological effects. Moreover, several COVID-19 mental health related studies have put more focus on evaluating its effect on the infected and high-risk groups such as people with comorbidities, old age, etc and less focus on other vulnerable groups such as young people, who have been proven to have a higher prevalence of psychological distress compared to other age groups before the pandemic [17]. Therefore, their levels psychological distress are expected to increase during the pandemic due to closure of schools, limited social interactions, missing several opportunities to further their studies and better their careers, future uncertainties, financial and economic strains, fears of infection of oneself and losing family members, among others [18, 19].

Although a few studies demonstrated the prevalence of mental health in university students during the pandemic, they did not demonstrate that different groups of young people have significantly different levels of psychological distress, depending on their country of citizenship or residence, government policies, economic status, healthcare systems, cultural norms, and other factors. This evaluation is important in finding the root causes of psychological distress in different groups in pursuance to applying specific mental health interventions and solutions with effectiveness. Moreover, comparing psychological distress levels and causative factors within different groups is important for learning strategies and interventions that were used and proven to be effective in the less affected groups.

COVID-19 psychologically affects University students in China and Africa differently, and factors including household income, households with relatives affected by COVID-19, religion, gender, educational background of household and age play a significant role in the differences. Given that, our research aims to investigate the prevalence of anxiety and depression in Chinese university students in China, African university students in China and African university students in Africa during the COVID-19 pandemic, to investigate the significant factors contributing to the prevalence of anxiety and depression, to evaluate the differences in factors affecting the different groups, and to emphasize that the psychological interventions are as important as the physical interventions during and after the pandemic.

## Method

### Study design and participants

To evaluate the psychological impact of COVID-19 on university students, a survey was created and distributed online from June 11, 2021, to September 27, 2021, to African university students in China, Chinese university students in China, and African university students in Africa. Prior to commencement, the Ethics Committee of Fujian Medical University Union Hospital and Fujian Medical University Experimental Animal Center reviewed and approved the study. All the participants were informed about the purpose and objectives of the study and voluntarily provided their verbal consent to participate. The survey data was kept with confidentiality and anonymity, and the study conformed to the ethical guidelines of the Declaration of Helsinki.

### Inclusion criteria

- Only Chinese and African university students could participate.
- Willingness to participate.

### Exclusion criteria

- Students who were not willing to be involved.
- Students with a history of psychiatric disorders.

The sample size of the study was determined using Cochran’s method of sample size estimation with a 0.05 margin of error. A 50% probability of satisfaction and 10% non-response rate were assumed and a suitable sample size was considered to be at least 200 participants per group [20]. Therefore, a total number of 684 questionnaires were distributed via emails, Wechat, WhatsApp, and Instagram, and the response rate was 92.98%.

### Data collection and instruments

The questionnaire consisted of three sections to gather information.

- Section one comprised of demographic information like age (<35 or ≥35), gender, country, education background (undergraduate, masters and PhD), faculty (medical or non-medical), religion (yes or no), monthly household income (<$500, $500–1000, $1000–5000 or >$5000), parents’ educational background (primary, secondary or tertiary), and family members/relatives who were infected with COVID-19 (yes or no).
- Section two comprised of the Patient Health Questionnaire PHQ-9, a diagnostic instrument widely used to evaluate the severity of depression in primary and mental health care. It is a 9 item depression scale, measured on four levels (not at all = 0, several days = 1, more than half days = 2, and nearly every day = 3), with participants reporting the frequency of symptoms experienced within the last two weeks. Severity is classified as minimal (0–4), mild (5–9), moderate (10–14), moderately severe (15–19), and severe (20–27) [1].
- Section three comprised of the Generalized Anxiety Disorder GAD-7, a screening questionnaire for measuring the severity of major anxiety disorders in mental health care. It is a 7 item anxiety scale with participants reporting the frequency of symptoms within the last two weeks. Severity is classified as minimal (0–4), mild (5–9), moderate (10–14), and severe (15–21) [21].

The PHQ-9 and GAD-7 diagnostic scales are widely used in psychology research [22, 23]. Their validity and reliability has been tested in several studies and demonstrated to have high internal consistency such as Cronbach’s α = 0.85 for PHQ-9 and Cronbach’s α = 0.91 for GAD-7 [24, 25].

### Data analysis

Measured values are given as a mean +/- standard deviation (SD). Statistical analysis was performed using SPSS for Windows Version 22. To compare the depression and anxiety scores and significant factors causing depression and anxiety in three groups of university students, Chinese students in China, African students in China and African students in Africa. T-test, one-way ANOVA (post-hoc Tukey test) were used. A p-value of less than 0.05 was considered statistically significant.

## Results

### General data

Of the 636 questionnaires collected, 222(34.9%) respondents were Chinese university students in China, 202(31.8%) were African university students in China and 212(33.3%) were African university students in Africa. Among the 636 participants, 97.5% were <35years; 57.3% were females; 72.2% were undergraduates, 24.4% were masters students, and 3.4% were Ph.D. students; 59.4% were non-medical majors; 32.7% were non-religious; 52.1% of parents had tertiary education background, 23.7% had secondary and 24.2% had primary; 21.5% earned <$500, 35.1% $500-$1000, 31.9% $1000-$5000 and 11.5%>$5000 monthly household income. Respondents with a family member affected by COVID-19 constituted 20.8% (Table 1).

**Table 1:** General demographics.

1. Variable	f	%
	N = 636	
2. Age(years) <35	620	97.5
≥35	16	2.5
3. Gender Female	364	57.3
Male	272	42.7
4. Groups Chinese in China	222	34.9
Africans in China	202	31.8
Africans in Africa	212	33.3
5. Education background Undergraduate	459	72.2
Master	155	24.4
PhD	22	3.4
6. Major Medical	258	40.6
Non-medical	378	59.4
7. Religion YES	428	67.3
NO	208	32.7
8. Parents’ Education Background Primary	154	24.2
Secondary	151	23.7
Tertiary	331	52.1
9. Monthly Household Income <$500	137	21.5
$500–1000	223	35.1
$1000–5000	203	31.9
>$5000	73	11.5
10. Relative with/had COVID19 YES	132	20.8
NO	504	79.2

### Depression

Based on the cut-off score of PHQ-9 which is 4, of the 636 respondents, 361 (56.8%) had depression. The prevalence of depression in the Chinese students in China group is 35.6% with a group mean of 4.06 (95% CI 3.36–4.77), Africans in China group is 70.3% with a group mean of 8.45 (95% CI 7.51–9.39) and Africans in Africa is 66.0% with a group mean of 8.71 (95% CI 7.78–9.64). According to the cross tab values (P<0.05), there is enough statistical evidence to show that there is a significant difference in depression levels between the Chinese group and the African groups (Africans in China and Africans in Africa) (Table 2).

**Table 2:** Prevalence of depression.

			Group			Total
			Chinese in China	Africans in China	Africans in Africa	
		**Total Count**	222	202	212	636
**Depression status**	**0 No**	**Count**	143_a_	60_b_	72_b_	275
		**% within Depression status**	64.4%	29.7%	34.0%	43.2%
	**1 Yes**	**Count**	79_a_	142_b_	140_b_	361
		**% within Depression status**	35.6%	70.3%	66.0%	56.8%
		**Total %**	100%	100%	100%	100%

Each subscript letter denotes a subset of group categories whose column proportions do not differ significantly from each other at the .05 level.

### Anxiety

Based on the cut-off scores for the GAD-7 which is 7, of the 636 respondents, 227 (35.7%) had anxiety. The prevalence of anxiety in the Chinese students in China group is 13.1% with a group mean of 3.37 (95% CI 2.84–3.91), the Africans in China group is 45.0% with a group mean of 8.05 (95% CI 7.24–8.86) and Africans in Africa is 50.5% with a group mean of 8.17 (95% CI 7.40–8.94). According to the cross tab values (P<0.05), there is enough statistical evidence to show that there is a significant difference in anxiety levels between the Chinese group compared to both African groups (Table 3).

**Table 3:** Prevalence of anxiety.

			Group			Total
			Chinese in China	Africans in China	Africans in Africa	
		**Total Count**	222	202	212	636
**Anxiety status**	**0 No**	**Count**	193_a_	111_b_	105_b_	409
		**% within Anxiety status**	86.9%	55.0%	49.5%	64.3%
	**1 Yes**	**Count**	29a	91b	107_b_	227
		**% within Anxiety status**	13.1%	45.0%	50.5%	35.7%
		**Total %**	100%	100%	100%	100%

Each subscript letter denotes a subset of group categories whose column proportions do not differ significantly from each other at the .05 level.

According to the Logistic regression data, country of citizenship (region), religion, parents’ educational background, household monthly income and, having family members with COVID-19, showed significant association with depression and anxiety in all groups compared together at P<0.05. In contrast, other demographic variables, age, gender, educational background and, major showed no significant association with depression and anxiety in all groups combined (Table 4).

**Table 4:** Descriptive analysis of association between sociodemographic characteristics and psychological state on all groups.

Variable			Depression		Depression		Anxiety		Anxiety	
	f	%	f	%	χ^2^	P-value	f	%	χ^2^	P-value
	N = 636									
**1. Age** <35	620	97.5	352	56.8	0.149	0.699	220	35.5	0.466	0.495
≥35	16	2.5	9	56.3			7	43.8		
**2. Gender** Female	364	57.3	152	41.8	0.002	0.967	134	36.8	0.464	0.496
Male	272	42.7	209	76.8			93	34.2		
**3. Region** China	222	34.9	79	35.6	62.309	**0.000**	29	13.1	76.085	**0.000**
Africa	414	63.1	142	70.3			91	45.0		
			140	66.0			107	50.5		
**4. Education background**										
Undergraduate	459	72.2	269	58.6	2.327	0.312	170	37.0	2.130	0.345
Master	155	24.4	81	52.3			48	30.1		
PhD	22	3.4	11	50.0			9	40.9		
**5. Major** Medical	258	40.6	136	52.7	2.898	0.089	81	31.4	3.491	0.062
Non-medical	378	59.4	225	59.5			146	38.6		
**6. Religion** YES	428	67.3	284	66.4	49.082	**0.000**	196	48.8	58.192	**0.000**
NO	208	32.7	77	37.0			31	14.9		
**7. Parent’s education background**										
Primary	154	24.2	69	44.8	11.846	**0.003**	23	14.9	42.268	**0.000**
Secondary	151	23.7	92	60.9			54	35.8		
Tertiary	331	52.1	200	60.4			150	45.3		
**8. Monthly Household Income**										
**<**$500	137	21.5	105	76.6	29.402	**0.000**	70	51.1	19.828	**0.000**
$500–1000	223	35.1	116	52.0			68	30.5		
$1000–5000	203	31.9	99	48.8			61	30.0		
>$5000	73	11.5	41	56.1			28	38.4		
**9. Relative with/had COVID19**										
YES	132	20.8	97	73.5	18.982	**0.000**	75	56.8	32.390	**0.000**
NO	504	79.2	264	52.4			152	30.2		

Comparing the three groups separately using chi square test, the variable statistically significant for both depression and anxiety in the Chinese in China group was major, for depression only was parent’s educational background and gender for anxiety only (Table 5).

**Table 5:** Descriptive analysis of association between sociodemographic characteristics and psychological state on the Chinese In China group.

Variable	f	%	Anxiety		Depression	
	N = 222		χ^2^	P-value	χ^2^	P-value
**1. Age** <35	218	98.1	0.612	0.434	0.369	0.543
≥35	4	1.9				
**2. Gender** Female	166	74.8	3.916	**0.048**	0.387	0.534
Male	56	25.2				
**3. Education Background**						
Undergraduate	158	71.2	0.964	0.618	0.258	0.867
Master	62	27.9				
PhD	2	0.9				
**4. Major** Medical	99	44.6	5.650	**0.017**	4.157	**0.041**
Non-medical	123	55.4				
**5. Religion** YES	31	14.0				
NO	191	86.0	1.982	0.159	0.634	0.426
**6. Parents’ Education Background**						
Primary	123	55.4	2.419	0.298	7.484	**0.024**
Secondary	47	21.2				
Tertiary	52	23.4				
**7. Monthly Household Income**						
<$500	0	0	0.313	0.855	0.547	0.761
$500–1000	106	47.7				
$1000–5000	96	43.3				
>$5000	20	9				
**8. Relative with/had COVID-19** YES	0	0				
NO	222	100	**-**	**-**	**-**	**-**

The variable statistically significant for both depression and anxiety in the Africans in China group was gender, and for anxiety only were religion and having family members with COVID-19 (Table 6).

**Table 6:** Descriptive analysis of association between sociodemographic characteristics and psychological state on the Africans In China group.

Variable	f	%	Anxiety		Depression	
	N = 202		χ^2^	P-value	χ^2^	P-value
**1. Age** <35	194	96.0	3.018	0.082	0.088	0.776
≥35	8	4.0				
**2. Gender** Female	76	37.6	6.543	**0.011**	9.261	**0.002**
Male	126	62.4				
**3. Education Background**						
Undergraduate	124	61.4	0.646	0.724	3.452	0.180
Master	59	29.2				
PhD	19	9.4				
**4. Major** Medical	92	45.5	0.482	0.487	0.268	0.605
Non-medical	110	54.5				
**5. Religion** YES	193	95.5				
NO	9	4.5	4.076	**0.043**	0.252	0.615
**6. Parents’ Education Background**						
Primary	18	8.9	2.828	0.243	2.423	0.298
Secondary	47	23.3				
Tertiary	137	67.8				
**7. Monthly Household Income**						
<$500	60	29.7	2.098	0.552	4.651	0.199
$500–1000	67	33.2				
$1000–5000	52	25.7				
>$5000	23	11.4				
**8. Relative with/had COVID-19** YES	48	23.8				
NO	154	76.2	7.745	**0.005**	2.372	0.124

The variable statistically significant for depression in the Africans in Africa group was educational background. There was no statistically significant variable for anxiety (Table 7).

**Table 7:** Descriptive analysis of association between sociodemographic characteristics and psychological state on the Africans In Africa group.

Variable	f	%	Anxiety		Depression	
	N = 212		χ^2^	P-value	χ^2^	P-value
**1. Age** <35	208	98.1	1.058	0.304	3.061	0.080
≥35	4	1.9				
**2. Gender** Female	122	57.5	0.906	0.341	1.693	0.193
Male	90	42.5				
**3. Education background** Undergraduate	177	83.5	2.498	0.287	6.785	**0.034**
Master	34	16.0				
PhD	1	0.5				
**4. Major** Medical	67	31.6	0.122	0.726	0.055	0.814
Non-medical	145	68.4				
**5. Religion** YES	204	96.2				
NO	8	3.8	2.158	0.142	0.953	0.329
**6. Parents’ Education Background** Primary	13	6.1	0.945	0.624	0.658	0.720
Secondary	57	26.9				
Tertiary	142	67.0				
**7. Monthly Household Income** <$500	77	36.3	3.057	0.383	4.192	0.241
$500–1000	50	23.6				
$1000–5000	55	25.9				
>$5000	30	14.2				
**8. Relative with/had COVID-19** YES	84	39.6				
NO	128	60.4	0.535	0.465	1.094	0.295

According to the chi squared tests at significance level (P<0.05), there is enough statistical evidence to show that there is a significant difference in depression and anxiety levels between the Chinese group compared to both African groups (showing similarly higher levels) (Table 8).

**Table 8:** χ^2^ test of significant differences in depression and anxiety levels between all groups.

	χ^2^	P-value
**Depression Chinese in China Africans in China**	51.065	0.000
** Chinese in China Africans in Africa**	40.228	0.000
** Africans in China Africans in Africa**	0.864	0.353
**Anxiety Chinese in China Africans in China**	53.327	0.000
** Chinese in China Africans in Africa**	70.529	0.000
** Africans in China Africans in Africa**	1.219	0.270

## Discussion

Previous studies have shown that public health epidemics can have psychological consequences on individuals, which can be expressed as depression, fear, anxiety, worry, and stress among others. University students are particularly considered a population vulnerable to mental health concerns [26]. Therefore, our research aims to investigate the prevalence of anxiety and depression in Chinese university students in China, African students in China and African students in Africa during the COVID-19 pandemic, to investigate the significant factors contributing to the prevalence of depression and anxiety, to evaluate the differences in factors affecting the different groups and to emphasize that the psychological interventions are as important as the physical interventions during and after the pandemic. We compared these groups in two ways i) individual groups all together and ii) individual groups separately.

In this paper, we discuss the hypothesis that COVID-19 affects university students at different levels due to factors like household income, having relatives with COVID-19, religion, gender, educational background and age.

### Factors causing depression and anxiety in individual groups all together

Our study demonstrated the prevalence of psychological distress on university students and the main stressors included; uncertainties of future careers, families’ financial losses, academic delays, quality of online classes, limited social interactions, fear of infection or loss of family members, etc [27] (Tables 2 & 3). The study also showed that African university students in China and African university students in Africa had significantly higher levels of depression and anxiety compared to Chinese university students in China (Tables 2, 3 & 8). The significant factors causing depression and anxiety within the groups were identified in our study as; country of citizenship (region), religion, parents’ educational background, household monthly income and, having family members with COVID-19 (Table 4). The Chinese students’ lower levels of depression and anxiety can be attributed to their country’s capability to manage the pandemic risk, determined by its stable economy, strong healthcare system and government policies. The effective management of the pandemic through i) provision of adequate healthcare facilities (building hospitals, mass production and quick distribution of testing kits, PPEs, vaccines, etc) ii) implementation of effective prevention strategies (quarantine, social distancing, universal body temperature checks, compulsory health QR codes, digital contact tracing, monitoring media reports, etc) and, iii) establishing mental health facilities and training students coping strategies in Chinese universities played an important role in lowering psychological harm in Chinese students [28–30].

In contrast, African students’ higher levels of depression and anxiety can be attributed to the countries’ incapability to manage the pandemic due to weak economies and healthcare systems [31–34]. Our study also found that students with higher family household income had lower levels of depression and anxiety as they could cater for their children’s basic needs, tuition and healthcare needs during the pandemic as supported by other studies [28, 35]. Most African families are large and have a monthly household income of <US$500 compared to most Chinese families which are generally small and about US$800-US$1500 family income, therefore African students had more psychological distress [36]. We also found that students whose parents had tertiary educational background had higher psychological distress than those with primary educational background.

Having a family member with COVID-19 caused high anxiety levels as supported by other studies [28, 37]. African students were mostly affected because of the fear of infection or death of their families due to the impracticability to implement effective prevention strategies to Africa along with the weak healthcare system.

### Factors causing depression and anxiety within the individual groups separately

#### Chinese university students in China group

Academic major (medical vs non-medical) was a significant factor causing depression and anxiety in this group with being a medical major having lower levels (Table 5). This is consistent with other studies that highlighted that medical students’ accurate knowledge of the disease’s causes, transmission, prevention and treatment is a protective factor of the psychological harm [38, 39]. Having parents with tertiary educational background significantly was also found to cause higher depression and this was inconsistent with other studies that demonstrated health literacy as a protective mental health factor [40, 41]. We anticipated parents with higher educational levels to have lower depression level as they have better knowledge about mental health problems hence more capable of identifying them and seeking help. Our findings also highlighted being female as another significant cause of higher anxiety scores in this group. This finding was supported by other studies [42–44], and is most likely due to their differential neuro-biological responses [45, 46] and, female university students generally having more stressful life events than males [47, 48].

#### African university students in China group

Gender was also a significant factor causing depression and anxiety in this group (Table 6). Unlike the Chinese females, most African young women have higher depression and anxiety levels most likely due to gender inequality in the academic, social and employment sectors, greater burden of housework, caregiving roles, risk of gender-based violence, cultural constraints, and the pressure of fulfilling societal standards and expectations on women [47–50]. Another factor we identified to be associated with high anxiety scores in this group was religion with religious people having higher anxiety levels. We expected religion to be a protective factor as it promotes faith, positive attitude, and mindful coping with mental health [51]. However, other studies showed that other religious beliefs interpreted COVID-19 as end times therefore some religious African university students in China were more anxious of end times without meeting their families [52, 53]. We also found that students with family members who had COVID-19 had significantly higher anxiety scores because of fear of infection and loss of their family members in Africa. Higher anxiety levels were attributed to feelings of hopeless and helpless in getting their families adequate healthcare protection during the pandemic as African countries have relatively weak economies and healthcare systems [54, 55], with overcrowded and understaffed hospitals and shortages in healthcare facilities [54, 55]. In addition, apart from healthcare facilities shortages, the thought of the impracticability of implementing prevention strategies effectively to their families caused more worry. For instance, social distancing is impracticable as many communities have large families residing in overcrowded housings, inaccessibility of clean water sources makes frequent hand washing nearly impossible, shortages and high costs of masks, etc [54, 56].

#### African university students in Africa group

Educational background was a significant factor causing high depression scores in Africans in Africa group where undergraduates were more affected than Ph.D students (Table 7). This can be due to senior undergraduates having greater workload, the pressure of facing graduation and looking for employment [57] in an economy significantly affected by the pandemic. In addition, unlike in China, Africa has a lack of health literacy which implies that higher education students have better knowledge about the disease and prevention measures therefore further protecting them from contracting the virus and it’s psychological impact [40]. There was no significant factor causing anxiety.

An unexpected finding was having Africans in China group and the Africans in Africa group with significantly similar levels of depression and anxiety (Tables 2, 3, and 8). We expected the Africans in China students to have lower levels because they benefited from the effective COVID-19 and mental health interventions implemented in China. However, our findings showed that having a family members with COVID-19 was a significant causative factor of anxiety as these students feared the infection/loss of family members back home due to uncertainties of the quality of protection and healthcare. Several other factors may have led to Africans in China having higher levels such as limited access to public resources due to informational, language, financial, and cultural barriers in China, loneliness, helplessness, lack of social support, inability to visit families, fear of not graduating on time, fear of economic collapse, and future job uncertainties. Another study also supported this finding stating that international students are one of the most affected groups and are easily neglected in pandemics especially if their home countries are also in a crisis [58, 59].

## Strengths and limitations

Identifying the significant factors causing depression and anxiety in the different student groups was the main strength of our research. It was vital to outline these differences in the level of depression and anxiety in these specific groups, enabling us to understand the causative factors behind our findings.

Our study also met some limitations. The mode of distribution of our questionnaire was through online platforms, this compromised access and quality of the results. Only students with access to the internet were the participants in our research. Since there was restriction of movement, collection of data was mainly online, thus participants were more prone to making errors when filling in information due to lack of strict supervision which may limit quality of results obtained. More effective ways of data collection such as one on one interviews can be implemented especially in the conditions being assessed, clinical symptoms and examinations can also be beneficial in understanding the depth of the condition. Furthermore, the sample size and selection was another limitation in our study which affected the following parameters; Age (Age was classified into two groups, <35 and >35 years old, majority of our participants were <35 years old which showed some bias of our results on evaluating this factor); Major (majority of participants were undergraduates); Religion (over 90% of religious people were from the African groups) and COVID-19 (Chinese group participants had no family members with COVID-19) which may also have limited the quality of results. Most of our samples were from one university town, the size and selection was hardly representative of all China and in the case of Africa only major cities had access to the study. A larger sample size would have evaluated and targeted more hard-hit provinces. Future studies can be improved by recruiting more participants from different regions with help from major governmental parties such as the ministry of education. In addition, lack of control groups for our study was a major setback. In the instance of a participant already affected by these mental disorders would affect responses they give on the survey as a result it compromises the quality of our results.

## Conclusion

Based on our findings, we can conclude that although university students are not COVID-19 high-risk groups, they were greatly psychologically affected by the pandemic. Among the groups in comparison, African students in China and African students in Africa had equally higher levels of anxiety and depression compared to the Chinese university students in China, each group showing specific factors significantly associated with depression and anxiety as discussed above.

According to our findings, University students are a COVID-19 mental health vulnerable group that requires immediate attention and psychological support. It is important that universities set up support groups and have professionals help with counseling to affected students. In China, universities started training students effective emotions controlling mechanisms during crisis and management of public health emergencies during the pandemic. These efforts by the Chinese government helped reduce mortality and morbidity, restore the confidence of protection of Chinese in China group and their families hence lowering mental distress. Whereas in Africa there is still stigma and discrimination, therefore it is important to educate people about mental health problems and their coping strategies. During lock down periods, the schools should arrange activities within the closed gates, to help with more student interactions. Continual reassurance of students about a better future and hope for disease alleviation should be a common theme in institutions. Failure to protect young people’s mental health and capitalize on their vitality may result in long-term loss of future productivity.

The government should allocate a budget to invest in mental health reforms and research, to help provide adequate mental care, building new hospitals and mental health facilities, mass production and quick distribution of health care facilities such as testing kits, PPEs, vaccines, etc to contain the virus. The government should also support the business sector through giving loans and lowering tax charges in order to minimize financial burdens. This helps minimize stress and uncertainty of the future in young entrepreneurs and also keep businesses afloat thus keeping students in school. As highlighted in this study, it is important to understand the specific causative factors of mental health in vulnerable groups to find the root causes and apply specific psychological interventions effectively. This suggests further similar investigations on other vulnerable groups. Further research can be done to identify other unknown COVID-19 mental health vulnerable groups. In addition, research to address how best these vulnerable groups can reduce mental health effects under pandemic conditions is vital.

## Supporting information

S1 Questionnaire Chinese in China. (PDF)

S2 Questionnaire African in China. (PDF)

S3 Questionnaire Africans in Africa. (PDF)

S1 File Final (defined entries) final. (XLSX)
