# Supplementary material for: The psychological impact of COVID-19 on university students in China and Africa
Source: PLoS One. 2022 Aug 4;17(8):e0270824. doi: 10.1371/journal.pone.0270824 (PMC9352056; doi:10.1371/journal.pone.0270824)
Supplement: S1 Questionnaire — (PDF) [file pone.0270824.s001.pdf]

研究旨在评估COVID-19对大学生的心理影响[复制][复制][复制]

这一次的疫情对大家带来了很多影响和困扰， 这个调查主要是研究疫情对大学生的心理影响。 请您用几分钟答以下的问题。非常感谢

1. 您的年龄是

- ○ 16-20
- ○ 21-30
- ○ 31-40

2. 您的性别

- ○ 男
- ○ 女

3. 您是哪里人? （省）

____

4.您的学历

- ○ 本科
- ○ 硕士
- ○ 博士

5. 您的专业

- ○ 医学专业
- ○ 非医学专业

6. 您父母的学历

- 高中以下
- 高中毕业
- 本科
- 硕士
- 博士

7. 家庭月收入 （家庭全部成员的收入）

- 5k-1w
- 1w-3w
- 3w-5w
- >5w

8. 您信仰宗教吗

- 信
- 不信

10. 有没有家人患过新冠肺炎?

- 有
- 没有

下面的问题是关于疫情之间 （0: 没有 1: 有几天 2: 一半以上 时间 3: 几乎天天）

11. 有没有感到不安、担心及烦躁

- 0
- 1

2

3

12. 有没有不能停止担心或控制不了担心

0

1

2

3

13. 有没有对各种各样的事情过度担心

0

1

2

3

14. 有没有很紧张 很难放松下来

0

1

2

3

15. 有没有感到非常焦躁以至无法静坐

0

1

2

3

16. 有没有变得容易烦恼或易被激怒

0

1

2

3

17. 感到好像有什么可怕的事会发生

0

1

2

3

18. 做事时提不起劲或没有兴趣

0

1

2

3

19. 感到心情低落,沮丧或绝望

0

1

2

3

20. 入睡困难、睡不安或睡得过多

- 0
- 1
- 2
- 3

21. 感觉疲倦或没有活力

- 0
- 1
- 2
- 3

22. 食欲不振或吃太多

- 0
- 1
- 2
- 3

23.觉得自己很糟或觉得自己很失败,或 让自己、家人失望

- 0
- 1
- 2
- 3

24. 对事物专注有困难,例如看报纸或看电视时

0

1

2

3

25. 行动或说话速度缓慢到别人已经察 觉?或刚好相反- 一变得比平日更烦 躁或坐立不安,动来动去

0

1

2

3

26. 有不如死掉或用某种方式伤害自己的念头

0

1

2

3

提交

举报

问卷星 提供技术支持
