# Supplementary material for: The psychological impact of COVID-19 on university students in China and Africa
Source: PLoS One. 2022 Aug 4;17(8):e0270824. doi: 10.1371/journal.pone.0270824 (PMC9352056; doi:10.1371/journal.pone.0270824)
Supplement: S3 Questionnaire — (PDF) [file pone.0270824.s003.pdf]

Evaluation of the psychological impact of COVID-19 on university students in Africa【复制】

Please take a few minutes to complete this survey. We value your feedback and your responses will be kept confidential.

---

1. Gender

- ○ Male
- ○ Female
- ○ Other

2. Age

- ○ 16-25 years
- ○ 26-35 years
- ○ > 35 years

3. Country

____

4. Educational Background

- ○ Undergraduate
- ○ Postgraduate
- ○ PhD

5. Major

- ○ Medical
- ○ Non-medical

6. Beliefs

- Christian
- Muslim
- Buddhism
- Non-religious
- Other

7. Parents’ Educational Background

- None
- Primary
- Secondary
- Tertiary

8. Economic Status (Family Income)

- < US$500 per month
- US$500- US$1000 per month
- US$1000- US$5000 per month
- > US$ 5000 per month

9. Family Member/Relatives with/had COVID-19

- Yes
- No

Please answer the following questions in reference to your experience during the COVID-19 period according to the following scales: (1=Not at all 2=Several days 3=More than half the days 4=Nearly everyday)

10. Feeling nervous, anxious or on edge

1 4

1 2 3 4

11. Not being able to stop or control worrying

1 4

1 2 3 4

12. Worrying too much about different things

1 4

1 2 3 4

13. Trouble relaxing

1 4

1 2 3 4

14. Being so restless that it is so hard to sit still

1 4

1 2 3 4

15. Becoming easily annoyed or irritated

1 4

1 2 3 4

16. Feeling afraid as if something off might happen

1 4

1 2 3 4

17. Little interest or pleasure in doing things

1 4

1 2 3 4

18. Feeling down, depressed or hopeless

1 4

1 2 3 4

19. Trouble falling/staying asleep, sleeping too much

1 4

1 2 3 4

20. Feeling tired or having little energy

1 4

1 2 3 4

21. Poor appetite or overeating

1 4

1 2 3 4

22. Feeling bad about yourself, or that you are a failure, or have let yourself, or your family down

1 4

1 2 3 4

23. Trouble concentrating on things, such as reading the newspaper or watching TV

1 4

1 2 3 4

24. Thoughts that you would be better off dead or of hurting yourself in some way

1 4

1 2 3 4

25. Moving or speaking so slowly that other people could have noticed or the opposite; being so fidgety or restless that you have been moving around more than usual

1 4

1 2 3 4

26. If any of the above problems were identified, how difficult have these problems made it for you to do your work, take care of things at home or get along with other people? (1= Not difficult 2= somewhat difficult 3= very difficult 4=extremely difficult）

1 4

1 2 3 4

Thank you for taking your time to complete this survey.

提交

举报

问卷星 提供技术支持
